# Balancing Glomerular Adequacy and Bleeding Risk in Native Kidney Biopsy: A Multicenter Cohort Study of Multiple Needle Passes

**DOI:** 10.1016/j.xkme.2026.101400

**Published:** 2026-05-12

**Authors:** Hisashi Kamido, Hiroki Mizuno, Yuki Oba, Shigekazu Kurihara, Masayuki Yamanouchi, Tatsuya Suwabe, Kei Kono, Kenichi Ohashi, Yoshifumi Ubara, Naoki Sawa

**Affiliations:** 1 Nephrology Center, Toranomon Hospital Kajigaya, Kanagawa, Japan; 2 Nephrology Center, Toranomon Hospital, Tokyo, Japan; 3 Okinaka Memorial Institute for Medical Research, Tokyo, Japan; 4 Department of Pathology, Toranomon Hospital, Tokyo, Japan; 5 Department of Human Pathology, Institute of Science Tokyo, Tokyo, Japan

**Keywords:** Needle passes, glomerular adequacy, major bleeding complications, hemoglobin decline, percutaneous kidney biopsy

## Abstract

**Rationale & Objective:**

Whether performing multiple needle passes during adult native kidney biopsy increases clinically significant bleeding is unclear. We evaluated the association between needle-pass number and major bleeding complications and hemoglobin level decline.

**Study Design:**

Multicenter retrospective cohort study.

**Setting & Participants:**

Adults undergoing percutaneous native kidney biopsy at 2 high-volume centers in Japan (January 2020 to December 2024) were included (n = 58). Open or lateral decubitus biopsies and cases with undocumented pass counts were excluded.

**Exposure:**

Number of needle passes.

**Outcomes:**

Major bleeding (transfusion, bladder irrigation, interventional radiology, surgical hemostasis, or death) and next-day hemoglobin level decline.

**Analytical Approach:**

Major bleeding was evaluated using Firth penalized logistic regression (unadjusted and estimated glomerular filtration rate adjusted). Hemoglobin level decline was evaluated using linear regression with a sensitivity analysis adjusting for baseline hemoglobin.

**Results:**

The median number of needle passes was 5 (interquartile range, 4-6). Glomerular adequacy (≥ 10 glomeruli) was achieved in 94.5%, with a median of 30 glomeruli (interquartile range, 17-52). Major bleeding occurred in 7 patients (1.5%), including transfusion in 3 (0.7%), bladder irrigation in 3 (0.7%), and interventional radiology in 1 (0.2%); no surgical hemostasis or deaths occurred. Needle-pass number was not significantly associated with major bleeding (odds ratio per pass, 1.23; 95% confidence interval [CI], 0.81-1.65; *P* = 0.29) or after adjustment for estimated glomerular filtration rate (odds ratio per pass, 1.14; 95% CI, 0.77-1.53; *P* = 0.46). Needle-pass number was not significantly associated with hemoglobin decline (β, −0.028 g/dL per pass; 95% CI, −0.065 to 0.009; *P* = 0.14), with consistent results in sensitivity analysis.

**Limitations:**

Major bleeding events were few, limiting precision; the retrospective 2-center design may limit generalizability and introduce bias.

**Conclusions:**

In this cohort, needle-pass number was not significantly associated with major bleeding or next-day hemoglobin decline. These findings support performing additional passes when needed to obtain adequate tissue while considering baseline bleeding risk.

## Plain Language Summary

A kidney biopsy is essential for diagnosing many kidney diseases, yet bleeding remains the most important complication. To obtain enough glomeruli for accurate interpretation, clinicians may take several tissue samples (“needle passes”). We studied 458 adults who underwent percutaneous native kidney biopsy at 2 high-volume hospitals in Japan between 2020 and 2024. The median number of passes was 5, producing a median of 30 glomeruli and adequate samples (at least 10 glomeruli) in 95% of biopsies. Major bleeding was uncommon (7 patients, 1.5%) and required transfusion, bladder irrigation, or radiology-based hemostasis; no one required surgery or died. A higher number of passes was not associated with major bleeding or with next-day hemoglobin level decline. These results support performing additional passes when needed to secure adequate tissue, while carefully considering baseline bleeding risks, especially in patients with reduced kidney function.

## Introduction

A kidney biopsy requires obtaining sufficient tissue for accurate diagnosis while minimizing complications, with bleeding being the most common and clinically significant adverse event.^1^ For light microscopic evaluation, 10 glomeruli are generally required for common diseases and 25 glomeruli for focal lesions.^1^^,^^2^ In recent years, inadequate sampling has become an increasing concern; the prevalence of inadequate samples in the United States kidney pathology registry increased from 2% in 2005 to 14% in 2020.^3^ Although 1-2 passes are generally recommended in pediatric kidney biopsies,^4^ there is no established recommendation regarding the number of passes in adults. Because severely reduced estimated glomerular filtration rate (eGFR) is associated with a higher likelihood of inadequate biopsy samples,^5^ a higher number of passes may be needed in adults to achieve an adequate glomerular count.

Bleeding complications after kidney biopsy have been evaluated in large-scale investigations, including national registry analyses, multicenter and single-center cohort studies, survey-based research, and systematic reviews across diverse regions.^6^, ^7^, ^8^, ^9^, ^10^, ^11^, ^12^, ^13^, ^14^ A systematic review and meta-analysis of studies worldwide was also published in 2020.^15^ Several factors have been associated with bleeding complications, including sex, age, anemia, thrombocytopenia, blood pressure, and antithrombotic therapy.^6^, ^7^, ^8^, ^9^, ^10^ However, across heterogeneous study designs and clinical settings, a low eGFR rate has been among the most consistently reported predictors of major bleeding.^6^, ^7^, ^8^, ^9^, ^10^

Only a limited number of studies have examined the association between the number of passes and bleeding complications,^8^^,^^11^ and findings have been inconsistent. Moreover, in these reports, the median number of passes was 1-2, and fewer than 5% of procedures involved ≥ 4 passes.

In routine practice at our institution, biopsy cores are assessed immediately, and the procedure is terminated once ≥ 10 glomeruli are confirmed, resulting in a higher number of passes than in prior studies. Therefore, this study aimed to evaluate the association between the number of needle passes and major bleeding complications and hemoglobin decline in adults undergoing percutaneous native kidney biopsy.

## Methods

### Study Setting and Participants

Patients who underwent percutaneous native kidney biopsy at 2 high-volume nephrology centers in Japan (Toranomon Hospital and Toranomon Hospital Kajigaya) between January 2020 and December 2024 were included. Exclusion criteria were open or lateral decubitus kidney biopsies and an undocumented number of needle passes.

All biopsies were performed during hospitalization with patients in the prone position, targeting the lower renal pole under real-time ultrasound guidance using an automated spring-loaded biopsy device (Acecut). Procedures were performed by nephrologists with 3-6 years of experience, ≥6 years of experience, or the department chief. A linear probe and a 16-gauge Acecut needle were used for all procedures.

Before biopsy, red blood cell transfusion was administered for a hemoglobin level ≤ 7 g/dL, and platelet transfusion was administered for a platelet count ≤ 20,000/μL. Antiplatelet agents were discontinued 7 days before the biopsy, and anticoagulants were discontinued 5 days before the biopsy with heparin bridging; heparin was stopped 6 hours before the procedure. The biopsy was terminated once ≥ 10 glomeruli were confirmed through immediate microscopic evaluation in the pathology laboratory; however, the procedure could be stopped earlier at the operator’s discretion for clinical reasons.

Postprocedure care included 15 minutes of manual compression, application of an abdominal binder, and strict bed rest for 24 hours. All patients received prophylactic oral antibiotics and tranexamic acid for 3 days. Antiplatelet agents and anticoagulants were resumed 24 hours after the biopsy. Routine postbiopsy computed tomography scan or ultrasound screening on the following day was not performed. Patients were observed in the hospital for at least 3 days and were followed up within 2 weeks after discharge.

Baseline demographic characteristics and laboratory data, kidney length at the time of biopsy, and pathology findings, including the total number of glomeruli, cortical tissue proportion (%), and pathologic diagnosis, were extracted from electronic medical records. The final glomerular count was determined by a pathologist using fixed tissue sections, and the average count across multiple slides was recorded.

### Association Between Needle Passes and Major Bleeding Complications

The occurrence of major bleeding complications was defined as bleeding requiring blood transfusion, bladder irrigation, interventional radiology, or surgical hemostasis, or resulting in death. Patients were compared according to the presence of major bleeding using the Mann-Whitney *U* test or the Fisher exact test. Given the low event rate, the association between the number of needle passes and major bleeding complications was evaluated using Firth penalized logistic regression.

### Association Between Needle Passes and Hemoglobin Level Decline

Hemoglobin level decline was defined as the hemoglobin level at the time of biopsy minus the hemoglobin level the following day. The association between the number of needle passes and hemoglobin level decline was assessed using linear regression. As a sensitivity analysis to account for baseline hemoglobin level, the hemoglobin level on the following day was modeled as the outcome with the baseline hemoglobin level and the number of needle passes included as predictors.

### Statistics and Software

All statistical analyses were conducted using R version 4.4.3 (R Foundation for Statistical Computing). Categorical variables are presented as numbers (percentages), and continuous variables are presented as medians (interquartile range [IQR]). Missing values in variables required for each analysis were handled by complete-case analysis. A 2-tailed *P*-value < 0.05 was considered statistically significant. This retrospective study was conducted in accordance with the principles of the Declaration of Helsinki. The study protocol was reviewed and approved by the Ethics Committee of Toranomon Hospital (Approval No. 2620-B). The requirement for informed consent was waived because of the retrospective design and use of anonymized data.

## Results

### Study Population and Biopsy Characteristics

A total of 458 patients were included (Fig S1). The median hemoglobin level at the time of biopsy was 13 g/dL (interquartile range, [IQR], 11-14), the median eGFR rate was 50 mL/min/1.73 m^2^ (IQR, 33-73), and the median number of needle passes was 5 (IQR, 4-6) (Table S1). Pathologic specimens contained a median of 30 glomeruli (IQR, 17-52) (Table S1). Glomerular adequacy (≥ 10 glomeruli) was achieved in 433 of 458 biopsies (94.5%). The most frequent diagnosis was immunoglobulin A nephropathy (n = 111, 24%), followed by nephrosclerosis (n = 72, 16%) and minor glomerular abnormality (n = 61, 13%) (Table S2).

### Association Between Needle Passes and Major Bleeding Complications

Major bleeding complications occurred in 7 patients (1.5%), including blood transfusion in 3 (0.7%), bladder irrigation in 3 (0.7%), and interventional radiology in 1 (0.2%). No patients required surgical hemostasis, and no deaths occurred. Compared with patients without major bleeding, those with major bleeding had a lower eGFR rate (37 mL/min/1.73 m^2^ [IQR, 15-55] vs 51 mL/min/1.73 m^2^ [IQR, 33-73]; *P* = 0.03) and lower hemoglobin (9 g/dL [IQR, 9-10] vs 13 g/dL [IQR, 11-14]; *P* < 0.01). There were no significant differences in the number of needle passes (6 [IQR, 4-7] vs 5 [IQR, 4-6]; *P* = 0.46) (Table 1).

**Table 1:** Patient Characteristics According to the Occurrence of Major Bleeding Complications

Characteristic	No Major Bleeding (n = 451)	Major Bleeding (n = 7)	*P*-value
Age, y	59 (45-71)	54 (42-67)	0.55
Male, n (%)	257 (57%)	4 (57%)	> 0.99
Height, cm	163 (157-170)	162 (156-168)	0.76
Weight, kg	60 (50-70)	56 (48-65)	0.75
BMI, kg/m^2^	22 (20-25)	22 (20-25)	0.84
sBP, mm Hg	127 (115-140)	139 (120-155)	0.25
dBP, mm Hg	77 (69-85)	80 (72-90)	0.33
Hemoglobin concentration, g/dL	13 (11-14)	9 (9-10)	0.01
Plt count, ×10^3^/μL	232 (188-275)	219 (190-245)	0.07
eGFR, mL/min/1.73 m^2^	51 (33-73)	37 (15-)55	0.03
APTT, sec	28 (26-31)	26 (23-29)	0.31
INR, ratio	1.0 (0.9-1)	1.0 (1.0-1.1)	0.91
Kidney length cm	9.4 (8.6-0)	9.0 (8.2-10)	0.78
Glomerular count, n	31 (21-52)	17 (14-36)	0.11
Cortical area, %	80 (65-90)	65 (50-80)	0.13
Needle passes, n	5 (4-6)	6 (4-7)	0.46

Note: Data are presented as median (interquartile range) or number (percentage). Between-group comparisons were performed using the Mann-Whitney *U* test or the Fisher exact test.

Abbreviations: APTT, activated partial thromboplastin time; BMI, body mass index; dBP, diastolic blood pressure; eGFR, estimated glomerular filtration rate; INR, international normalized ratio; Plt, platelet; sBP, systolic blood pressure.

In Firth penalized logistic regression, the number of needle passes was not significantly associated with major bleeding (odds ratio [OR] per pass, 1.23; 95% confidence interval [CI], 0.81-1.65; *P* = 0.29). After adjustment for eGFR rate, results were similar (odds ratio per pass, 1.14; 95% CI, 0.77-1.53; *P* = 0.46), whereas a higher eGFR rate was associated with lower odds of major bleeding (odds ratio per 10 mL/min/1.73 m^2^, 0.69; 95% CI, 0.45-0.97; *P* = 0.03) (Table 2).

**Table 2:** Association Between the Number of Needle Passes and Major Bleeding Complications (Firth Penalized Logistic Regression)

	OR (95% CI)	*P*-value
Univariable model Needle passes	1.23 (0.82-1.66)	0.29
Multivariable model Needle passes	1.14 (0.77-1.53)	0.46
eGFR (per 10 mL/min/1.73 m^2^)	0.70 (0.45-0.97)	0.03

Note: Results are presented as odds ratios with 95% CIs.

Abbreviations: CI, confidence interval; eGFR, estimated glomerular filtration rate; OR, odds ratio.

### Association Between Needle Passes and Hemoglobin Level Decline

Hemoglobin level decline was not significantly associated with the number of needle passes (β, −0.028 g/dL per pass; 95% CI, −0.065 to 0.009; *P* = 0.14). In a sensitivity analysis accounting for baseline hemoglobin, the number of needle passes was not significantly associated with hemoglobin on the following day (β, 0.013 g/dL per pass; 95% CI, −0.024 to 0.050; *P* = 0.49) (Table 3).

**Table 3:** Association Between the Number of Needle Passes and Postbiopsy Hemoglobin Level Change: Primary And Sensitivity Analyses

	β (95% CI)	*P* -value
Primary analysis Hemoglobin level decline	−0.028 g/dL per pass (−0.065 to 0.009)	0.14
Sensitivity analysis of hemoglobin level on the following day	0.013 g/dL per pass (−0.024 to 0.050)	0.49

Note: Results are presented as regression coefficients (β) with 95% CIs. The primary analysis modeled hemoglobin level decline (the baseline hemoglobin level minus the hemoglobin level on the following day) as the outcome. The sensitivity analysis modeled the hemoglobin level on the following day as the outcome and adjusted for baseline hemoglobin level. Abbreviations: CI, confidence interval.

## Discussion

In this multicenter retrospective cohort study of 458 adults undergoing percutaneous native kidney biopsy, the number of needle passes was not significantly associated with major bleeding complications or next-day hemoglobin level decline.

The median number of passes was 5, which is higher than in prior studies.^8^^,^^11^ Only 5.5% of biopsies did not meet the adequacy threshold of ≥ 10 glomeruli, which is lower than the 14% reported in the US Renal Pathology Registry^3^ and comparable with other high-quality series.^8^ All biopsies were performed using a 16-gauge needle, which has been shown to provide adequate tissue while maintaining a favorable safety profile.^15^ The distribution of underlying pathologic diagnoses in this cohort was largely consistent with previous reports.^11^ Notably, cases classified as “undefined nephropathy” were not observed, which may be attributable to the relatively high glomerular yield (median, 30 glomeruli), sufficient to detect both common diseases and focal lesions.^1^^,^^2^

Major bleeding complications occurred in 7 patients (1.5%), including transfusion in 3 (0.7%), bladder irrigation in 3 (0.7%), and interventional radiology in 1 (0.2%). These rates were similar to or lower than those in previous reports.^6^, ^7^, ^8^, ^9^, ^10^, ^11^, ^12^, ^13^, ^14^, ^15^ Prior work has suggested that a higher number of passes may increase the volume of hematoma detected on postbiopsy imaging^16^; however, most postbiopsy hematomas are small and of uncertain clinical significance.^15^ Consistent with these observations, the number of needle passes was not significantly associated with major bleeding complications in this cohort.

In addition to major bleeding, no association was observed between the number of needle passes and hemoglobin level decline on the following day. The sensitivity analysis accounting for baseline hemoglobin level yielded similar findings, supporting the robustness of the primary analysis and suggesting that additional passes did not result in clinically meaningful short-term loss of hemoglobin concentration in this setting.

Several factors have been linked to postbiopsy bleeding, but many are optimized periprocedurally, whereas a low eGFR rate has remained among the most consistently reported predictors.^6^, ^7^, ^8^, ^9^, ^10^ Consistent with prior reports, a lower eGFR rate was associated with higher odds of major bleeding in this cohort.

This study has several limitations. First, the small number of major bleeding events limited statistical power and precision. Second, the retrospective design may introduce selection and information biases, and findings from 2 high-volume centers in Japan may not be generalizable to other settings. Third, operator experience was not included. However, a prior report suggests that kidney biopsy can be performed with comparable complication rates and tissue yield across experience levels when conducted under a supervised training structure,^17^ which was also the procedural context in the present study. Despite these limitations, the study provides contemporary evidence from routine clinical practice in which multiple passes were frequently performed with immediate assessment of tissue adequacy.

In conclusion, the number of needle passes was not significantly associated with major bleeding complications or hemoglobin level decline. These findings support performing additional passes when needed to obtain adequate tissue, while carefully considering baseline bleeding risk.
