# Supplementary material for: Balancing Glomerular Adequacy and Bleeding Risk in Native Kidney Biopsy: A Multicenter Cohort Study of Multiple Needle Passes
Source: Kidney Med. 2026 May 12;8(7):101400. doi: 10.1016/j.xkme.2026.101400 (PMC13241847; doi:10.1016/j.xkme.2026.101400)

Supplementary materials

Table S1. Patient characteristics at the time of native kidney biopsy.
Table S2. Pathologic diagnoses in the study cohort.
Figure S1. Study flow diagram.

Table S1. Patient characteristics at the time of native kidney biopsy.

	**Overall (n=458)**
**Age [years]**	59 [45, 71]
**Male [n]**	261 (57%)
**Height [cm]**	163 [157, 170]
**Weight [kg]**	60 [50, 70]
**BMI [kg/m²]**	22 [20, 25]
**sBP [mmHg]**	127 [115, 140]
**dBP [mmHg]**	77 [69, 85]
**Hemoglobin [g/dL]**	13 [11, 14]
**Plt [$\times 10^3/\mu L$]**	232 [188, 275]
**eGFR [mL/min/1.73 m²]**	50 [33, 73]
**APTT [sec]**	28 [26, 31]
**INR [ratio]**	1.0 [0.9, 1.1]
**Kidney Length [cm]**	9.4 [8.6, 10]
**Glomerular count [n]**	30 [17, 52]
**Cortical area [%]**	80 [65, 90]
**Needle passes [n]**	5 [4, 6]

Note: Data are presented as median (interquartile range) or number (percentage).
Abbreviations: BMI, body mass index; sBP, systolic blood pressure; dBP, diastolic blood pressure; Plt, platelet count; eGFR, estimated glomerular filtration rate; APTT, activated partial thromboplastin time; INR, international normalized ratio.

Table S2. Pathologic diagnoses in the study cohort.

	n	%
**IgA nephropathy**	111	24
**Nephrosclerosis**	72	16
**Minor glomerular abnormalities**	61	13
**Diabetic nephropathy**	32	7
**Interstitial fibrosis and tubular atrophy**	23	5
**Membranous glomerulonephritis**	21	5
**Thrombotic microangiopathy**	18	4
**Lupus nephritis**	18	4
**Tubulointerstitial nephritis**	15	3
**Mesangioproliferative glomerulonephritis**	15	3
**Focal segmental glomerulosclerosis**	14	3
**Others**	58	13

Figure S1. Study flow diagram.

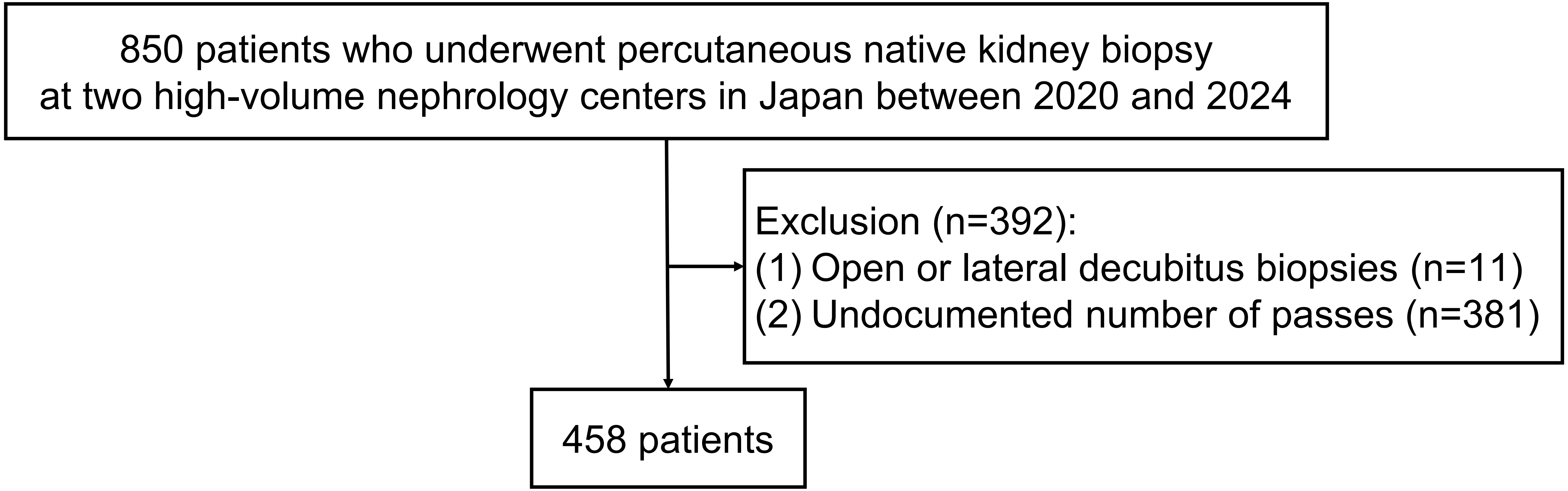

Supplement: Supplementary File (PDF) — Figure S1; Tables S1-S2 [file mmc1.pdf]
